# The effect of cardiac shock wave therapy on myocardial function and perfusion in the randomized, triple-blind, sham-procedure controlled study

**DOI:** 10.1186/s12947-019-0163-1

**Published:** 2019-07-04

**Authors:** Jelena Čelutkienė, Greta Burneikaitė, Evgeny Shkolnik, Gabrielius Jakutis, Donatas Vajauskas, Kamilė Čerlinskaitė, Gitana Zuozienė, Birutė Petrauskienė, Roma Puronaitė, Renata Komiagienė, Irena Butkuvienė, Rima Steponėnienė, Jonas Misiūra, Aleksandras Laucevičius

**Affiliations:** 1 Institute of Clinical Medicine of the Faculty of Medicine of Vilnius University, Santariskiu St. 2, 08661 Vilnius, Lithuania; 2 Yale-New Haven Health Bridgeport Hospital, 267 Grant St, Bridgeport, 06610 CT USA

**Keywords:** Cardiac shock wave therapy, Coronary artery disease, Stable angina, Myocardial perfusion, Dobutamine stress echocardiography, Single photon emission computed tomography

## Abstract

**Background:**

Recent triple-blind sham procedure-controlled study revealed neutral effects of the cardiac shock wave therapy (CSWT) on exercise tolerance and symptoms in patients with stable angina. Current data about the effects of CSWT on global and regional myocardial contractility and perfusion is limited. Hereby we report the results of an imaging sub-study that evaluated the capacity of CSWT to ameliorate myocardial ischemia induced during dobutamine stress echocardiography (DSE) and cardiac single photon emission computed tomography (SPECT).

**Methods:**

Prospective, randomized, triple-blind, sham procedure-controlled study enrolled 72 adult subjects who complied with defined inclusion criteria. The subjects were assigned to the OMT + CSWT and the OMT + sham procedure study groups with 1:1 ratio. Application of the CSWT covered all segments of the left ventricle. Imaging ischemia tests were performed in 59 study patients: DSE and SPECT before the CSWT treatment and after 6 months, with DSE carried out additionally at 3 months after randomization. Co-primary endpoints of the study were: change in wall motion score index (WMSI), representing the stress-induced impairment of regional myocardial function, and change in summed difference score (SDS), representing the amount of perfusion defect.

**Results:**

OMT + CSWT and OMT + sham procedure study groups included 30 and 29 patients, respectively. Regional myocardial contractility during DSE significantly improved at 3 months follow-up in OMT + CSWT group compared to baseline as shown by WMSI at stress (1.4 ± 0.4 vs 1.6 ± 0.4, *p* = 0.001), but not in OMT + sham procedure group (1.5 ± 0.3 vs 1.6 ± 0.4, *p* = 0.136). The difference in stress DSE results between both study groups disappeared after 6 months. SPECT results demonstrated a significant reduction of inducible ischemia in OMT + CSWT group compared to OMT + sham procedure group at 6 months follow-up (SDS dropped from 5.4 ± 3.7 to 3.6 ± 3.8 vs 6.4 ± 5.9 to 6.2 ± 5 respectively, *p* = 0.034).

**Conclusions:**

Cardiac shock wave treatment showed the ability to reduce stress-induced myocardial ischemia, as assessed by wall motion abnormalities and perfusion defects, compared to sham procedure.

**Trial registration:**

Clinicaltrials.gov (NCT02339454). The trial was registered retrospectively on 12 January 2015.

## Background

Major advances in medical therapy and revascularization techniques have markedly improved the quality of life of coronary artery disease (CAD) patients. However despite this progress, up to 14% of patients still face considerable symptomatic burden of refractory angina, which is not amenable to traditional revascularization options [1, 2].

Cardiac shock wave therapy (CSWT) is a newly developed method that utilizes a non-invasive application of low-intensity shock waves (SW), which induce the release of angiogenic factors such as endothelial nitric oxide synthase, vascular endothelial growth factor, and proliferating cell antinuclear antigen [3–5]. A number of published clinical studies showed the efficacy and safety of CSWT in patients with refractory angina [6–14]. Despite encouraging results, evidence supporting the efficacy of CSWT mostly come from small, uncontrolled, low to moderate quality single centre observational studies [15]. Moreover, only limited information about the effects of CSWT on global and regional myocardial contractility and perfusion is available in current literature. In addition to the prospective, randomized, triple blind, sham-procedure controlled trial [16], we performed an imaging sub-study to evaluate the capacity of CSWT to reduce the objective signs of myocardial ischemia, that were determined by dobutamine stress echocardiography (DSE) and SPECT.

## Methods

### Main study design

A prospective, randomized, triple blind, sham-procedure controlled study was designed to assess the antianginal efficacy of CSWT, on top of standard medical therapy in patients with stable angina. Study protocol was created according to Consolidated Standards of Reporting Trials (CONSORT) statement recommendations for parallel group randomized trials [17] and the study conducted in accordance with Good Clinical Practice, Declaration of Helsinki 2013. The design, methods, and results of the main trial (NCT02339454) were described previously [16, 18].

Briefly, patients diagnosed with angiography confirmed-CAD and exercise induced-angina associated with ST-segment depression ≥1 mm on treadmill electrocardiogram (ECG), and symptoms not controlled by optimal medical treatment (OMT), were enrolled in the study. Exclusion criteria were: angina at rest, acute coronary syndrome or planned coronary revascularization within 6 months, New York Heart Association (NYHA) heart failure class III-IV, thrombus in left ventricle, contraindications for exercise testing, ECG abnormalities at rest. Eligible subjects were assigned to the OMT + CSWT and the OMT + sham procedure study groups with 1:1 ratio. Patients, investigators (clinicians and data assessors), and a statistician were blinded to treatment allocation.

### Main study treatment

All patients were maintained on stable doses of medications [19] for 4 weeks before the baseline evaluation and the entire study period. CSWT was performed using Cardiospec device (Medispec Ltd., Germantown, Maryland, USA) coupled with a cardiac ultrasound imaging system (Vivid I; GE Healthcare, Horten, Norway) to target the treatment area under ECG R-wave gating. Treatment consisted of 9 sessions with 3 sessions per week and was performed on the first, fifth, and the ninth study weeks. Treatment intensity was equal to 100 impulses applied to one spot with up to 1200 impulses to the patient per session. During the first, fifth, and the ninth study weeks, SWs were delivered to the basal, middle, and apical segments of the left ventricle (LV), respectively, covering the whole LV [18].

### Imaging sub-study design

The imaging sub-study was conducted at Vilnius University Hospital Santaros klinikos (Vilnius, Lithuania) and was approved by Vilnius Regional Ethics Committee (Approval No. 158200–13–616-187). We hypothesized that compared to sham procedure, CSWT on top of the OMT will significantly reduce the stress-induced myocardial ischemia as detected by the ventricular wall motion and perfusion imaging tests.

The co-primary endpoints of the study were the anticipated change of:

1. stress wall motion score index (WMSI), representing the stress-induced impairment of regional myocardial function during DSE;
2. summed difference score (SDS), representing the amount of perfusion defect during SPECT.

The secondary endpoints included the anticipated changes of:

1. stress wall motion score and LVEF during DSE,
2. number of patients with at least moderate stress-induced ischemia,
3. frequency of angina and ST depression during DSE,
4. global and single-view systolic longitudinal strain,
5. stress summed score and total perfusion defect detected by SPECT.

Each sub-study patient underwent DSE and SPECT before the CSWT treatment and at 6-months follow-up, with DSE performed additionally at 3 months. Beta-blocking medications were discontinued for 48 h, other antianginal medications for 24 h prior to stress tests as recommended in Stress Echocardiography Expert Consensus Statement [20] and European Association of Nuclear Medicine procedural guidelines [21]. Analysis of each DSE and SPECT study images were performed by two independent observers blinded to the study data using the LV 17-segment model [22, 25, 26]. Discordant assessments were jointly reviewed.

### Dobutamine stress echocardiography

Dobutamine was infused at 5, 10, 20, 30, and 40 μg/kg/min. If no end point was reached, atropine (up to 1 mg) was added to the continuing 40 μg/kg/min dobutamine infusion.

Transthoracic stress echocardiographic studies were performed with a commercially available ultrasound machine (Vivid 7 and 9, GE Healthcare, Horten, Norway) with a 1.5–4.6 MHz transducer. Long and short axes of the LV from the parasternal window and 4-, 3- and 2-chamber views from the apical window were acquired for comparison in four stages of stress test. Images were stored digitally and analysed off-line using customised software (Echopac PCBT08, GE Healthcare). Segmental wall motion was semi-quantitatively graded as follows: normal = 1; hypokinetic, meaning marked reduction of endocardial motion and thickening = 2; akinetic defined as virtual absence of inward motion and thickening = 3; and dyskinetic, corresponding to paradoxic wall motion away from the centre of the LV in systole = 4. The sum of all segment scores generates wall motion score (WMS), which, when divided by the number of interpretable segments makes wall motion score index (WMSI).

Test positivity was defined as the occurrence of at least one of the following conditions: 1) new dyssynergy in a region with normal resting function (i.e., normokinesis becoming hypo-, aki- or dyskinetic), 2) worsening of a resting dyssynergy (i.e., a hypokinesia becoming aki- or dyskinesia). For dobutamine stress echocardiography evaluation, moderate ischemia was defined as ≥3 segments with stress induced severe hypokinesis or akinesis [23].

### Deformation imaging during DSE

Apical 2-, 3- and 4-chamber cine-loops for speckle tracking analysis were recorded at baseline and peak dobutamine levels with breath holding in the range of 70–90 frames/sec. After manual tracing of endocardium borders of the 2D images, the software automatically tracked myocardial motion, creating 6 regions of interest in each apical image, with tracking quality labelled as verified or unacceptable. In segments with unacceptable tracking, observer readjusted the endocardium trace line until a verified tracking was achieved. If this was not attainable, that segment was excluded from the analysis. Peak longitudinal global systolic and single 4-, 3- and 2- chambers view strains at rest and during stress were measured.

DSE analysis included WMSI, myocardial strain analysis and LV ejection fraction (EF) measured by Simpson’s biplane method.

### Myocardial perfusion imaging by SPECT

During SPECT stress was induced by infusion of adenosine at a standard rate of 140 μg/kg/min (maximal infusion duration of 6 min) [24]. A 1-day ECG gated stress and rest SPECT protocol was used. After 3 min of adenosine infusion patients were injected intravenously with a body mass index adjusted dose (250–350 MBq) of technetium 99 m (^99m^Tc)-sestamibi (MIBI). At-rest myocardial perfusion imaging (MPI) was performed at the same day, 4 h after the stress MPI, with identical acquisition protocol 60 min after ^99m^Tc-MIBI injection, with a dual-head INFINIA GP3 (GE Medical Systems, Waukesha, WI, USA) gamma camera.

Gated and non-gated SPECT MPI image sets were reconstructed using OSEM iterative reconstruction, with the dedicated Xeleris 2.1 workstation, using Cedars-Sinai QGS/QPS software package (Cedars-Sinai, Los Angeles, CA, USA). Each segment was scored separately using a 5-point model as follows: 0 = normal perfusion, 1 = minimal perfusion defect, 2 = moderate perfusion defect, 3 = severe perfusion defect, 4 = no perfusion. The variables included summed rest score (SRS), summed stress score (SSS), and summed difference score (SDS: stress minus rest score). Total perfusion defect (TPD) was calculated by dividing the summed scores by 68, which is the maximal potential score (4 × 17) and multiplying by 100. Reversible from stress to rest perfusion defects were considered to represent myocardial ischemia [21, 25, 26]. Summed difference score of 0 corresponds to normal perfusion, 1–4 to mild ischemia, 4–7 to moderate ischemia, and more than 7 as severe ischemia [27]. In one patient image quality was not amenable for interpretation, therefore his test was excluded from the analysis.

### Statistical analysis and sample size calculation

Baseline patients’ characteristics were descriptively summarized: continuous variables were expressed as mean value ± standard deviation (SD), whereas categorical variables were expressed as absolute number (percentage). Paired parameters were tested for normal distribution with the Shapiro-Wilk test. Chi-square tests or Fisher exact test were used to compare categorical variables. Difference between groups for variables with normal distribution was analysed by using parametric t-test, while for not normally distributed variables a non-parametric Mann-Whitney test was used. Wilcoxon signed rank test was used to compare paired data at baseline and follow-up.

*P* values < 0.05 (two sided) were considered statistically significant. The overall effect of the CSWT was evaluated by comparing the average change of variable in the treatment group with the average change of variable in the sham procedure group. Statistical analyses were performed with SPSS 20.0 (SPSS, Chicago, IL, USA).

For the sample size estimation, a power of 90% and a two-sided type I error of 5% were chosen. On the basis of study results [7], assuming a standard deviation of 6.4 for wall motion score, 22 patients per group were necessary to detect a ≥ 3 points difference. Estimating a withdrawal of 10% of patients after randomization, approximately 50 patients were needed to be included in the study. Assuming a standard deviation of 3.8 for summed difference score, 18 patients per group were necessary to detect a ≥ 3 points difference. Estimating withdrawal of 10% of patients after randomization, approximately 40 patients were needed to be included in the study.

### Inter-observer agreement

Inter-observer agreement of the DSE and SPECT evaluations was assessed by two independent investigators, who had measured the representative parameters of stress tests in 15 and 30 randomly selected patients, respectively. Reproducibility was expressed as the mean difference and the SD of the differences between values of observer 1 and observer 2 [28]. As a measure of reliability, intraclass correlation coefficients (ICC) and their 95% confidence intervals (CI) based on consistency of 2-way mixed effects model were calculated for each parameter using the icc{irr} function of R package (version 3.4.1) [29]. ICC values less than 0.5, 0.5–0.75, 0.75–0.9 and > 0.9 indicate poor, moderate, good and values excellent reliability, respectively [30].

## Results

From June 2013 to December 2015, 72 patients were randomized (1:1) in the main study, of them 59 underwent imaging tests: 30 patients entered the OMT + CSWT group and 29 patients were allocated to the OMT + sham procedure group. Complete data of the DSE were available in 28 patients of each group at 3-months follow-up, in 28 patients of OMT + CSWT group and 26 patients of OMT + sham procedure group at 6-months follow-up. Full data of myocardial perfusion imaging by SPECT were available in 26 and 25 patients of OMT + CSWT and OMT + sham procedure group, respectively. Each sub-study patient had positive either DSE or SPECT, the majority had both tests positive.

Baseline characteristics of the sub-study groups are presented in Table 1. Majority of patients (78%) had multivessel disease and 96% were not candidates for further revascularization due to the extent and the severity of the disease or technical considerations. Exercise capacity was moderately reduced in both study groups (total exercise duration in minutes was 6.2 ± 2.2 and 5.8 ± 2.2 in the OMT + sham and in the OMT + CSWT group, respectively, *p* = 0.558).

**Table 1:** Baseline characteristics of the study patients

Variable	OMT + CSWT group (*n* = 30)	OMT + sham procedure group (*n* = 29)	*P* value
Demographic characteristics			
Age, years	67.2 ± 7.8	69.4 ± 7.8	0.274
Male sex, n (%)	19 (63.3)	26 (89.7)	**0.018**
Cardiovascular risk factors			
Hyperlipidaemia, n (%)	30 (100)	29 (100)	–
Hypertension, n (%)	29 (96.7)	29 (100)	–
Diabetes, n (%)	8 (26.7)	8 (27.6)	0.937
Peripheral vascular disease, n (%)	10 (33.3)	12 (41.4)	0.523
Current smoker, n (%)	1 (3.3)	4 (13.8)	0.195
Positive family history for CVD, n (%)	10 (33.3)	19 (5.5)	**0.013**
Medical history			
Previous myocardial infarction, n (%)	15 (50.0)	23 (79.3)	**0.019**
Previous percutaneous intervention, n (%)	16 (53.3)	15 (51.7)	0.902
Previous CABG, n (%)	20 (66.7)	18 (62.1)	0.712
No revascularization, n (%)	7 (23.3)	7 (24.1)	0.936
Three-vessel disease, n (%)	24 (80.0)	22 (75.9)	0.161
Two-vessel disease, n (%)	5 (16.7)	2 (6.9)	
Clinical parameters			
Body mass index, kg/m^2^	30.0 ± 4.3	30.3 ± 3.8	0.755
Angina episodes/week, median (25%;75%)	5.5 (3.3; 14.8)	7 (3.8; 15)	0.500
Nitroglycerine consumption (times/week), median (25%;75%)	2 (1; 2)	2 (0; 5)	0.250
Left ventricular ejection fraction (echocardiographic), %	54.4 ± 9.5	56.0 ± 7.2	0.366
Systolic blood pressure, mmHg	124.7 ± 20.9	128.1 ± 22.1	0.845
Diastolic blood pressure, mmHg	81.1 ± 11.3	77.0 ± 11.2	0.341
Angina CCS class			
II, n (%)	8 (26.7)	9 (31.0)	0.711
III, n (%)	22 (73.3)	20 (69.0)	
Medical treatment			
ACE inhibitors / ARB, n (%)	30 (100)	29 (100)	–
Beta-blocker, n (%)	28 (93.1)	27 (93.1)	1
Long acting nitrates, n (%)	19 (63.3)	15 (51.7)	0.367
Calcium channel blocker, n (%)	16 (51.3)	15 (51.7)	0.902
Trimetazidine, n (%)	20 (66.7)	15 (51.7)	0.243
Statins, n (%)	30 (100)	29 (100)	–
Mean dose of atorvastatin, mg	36.2 ± 11.8^a^	40.3 ± 17.0	0.286
Antiplatelets, n (%)	30 (100)	29 (100)	–
Dual-antiplatelet therapy, n (%)	4 (13.3)	11 (37.9)	0.031
Oral anti-diabetics, n (%)	4 (13.3)	7 (24.1)	0.287
ECG Exercise test			
Exercise duration, sec	350.1 ± 133.1	370.4 ± 131.0	0.558

*CABG* Coronary artery bypass grafting, *CCS* Canadian Cardiovascular Society, *CSWT* Cardiac shock wave therapy, *CVD* cardiovascular disease, *OMT* optimal medical therapy, *SAQ* Seattle Angina Questionnaire

^a^one patient in this group was on fluvastatin 80 mg, not included in mean dose calculations

### Co-primary endpoints of the study

**Fig. 1:**
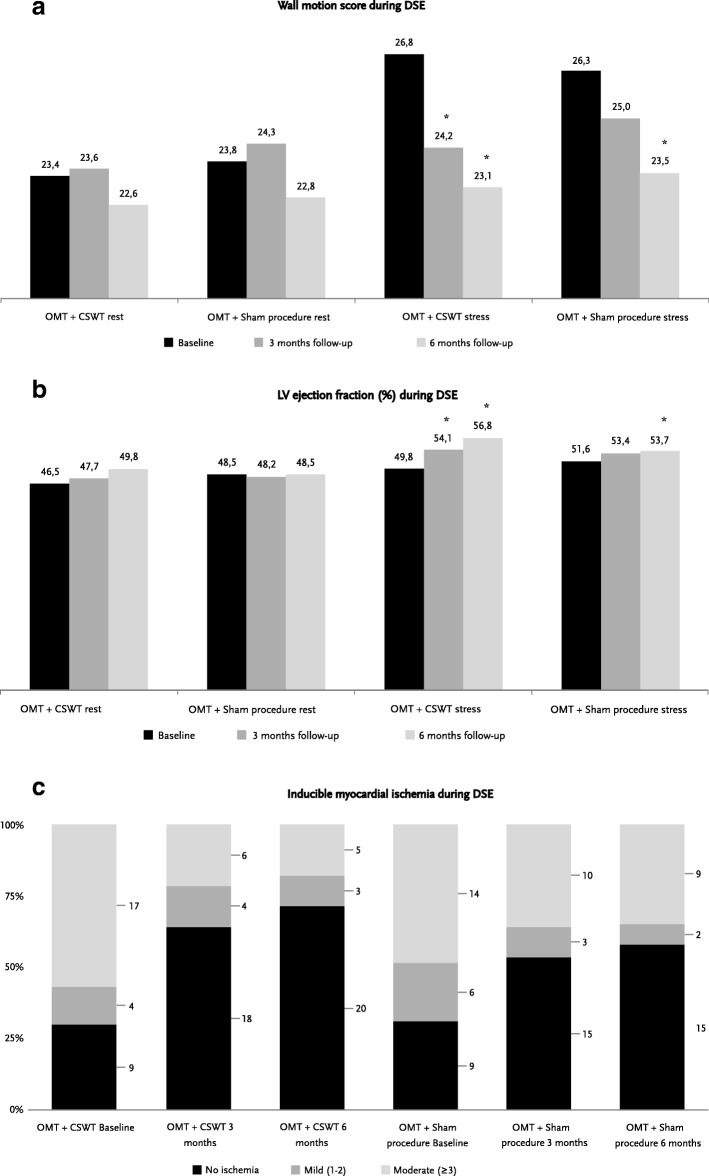
The dynamics of myocardial function and inducible ischemia evaluated by dobutamine stress echocardiography. **a** dynamics of wall motion score at baseline, 3 and 6 months of follow up in CSWT and sham procedure group; **b** changes of LV ejection fraction at baseline, 3 and 6 months of follow up in CSWT and sham procedure group; **c** distribution of mild, at least moderate or no ischemia at baseline, 3 and 6 months of follow up in CSWT and sham procedure group. CSWT – Cardiac shock wave therapy, LV – left ventricle, OMT – optimal medical therapy. Moderate ischemia defined as ≥3 segments with stress induced severe hypokinesis or akinesis. * - P was paired in the group and considered as significant (*P* < 0.05)

**Fig. 2:**
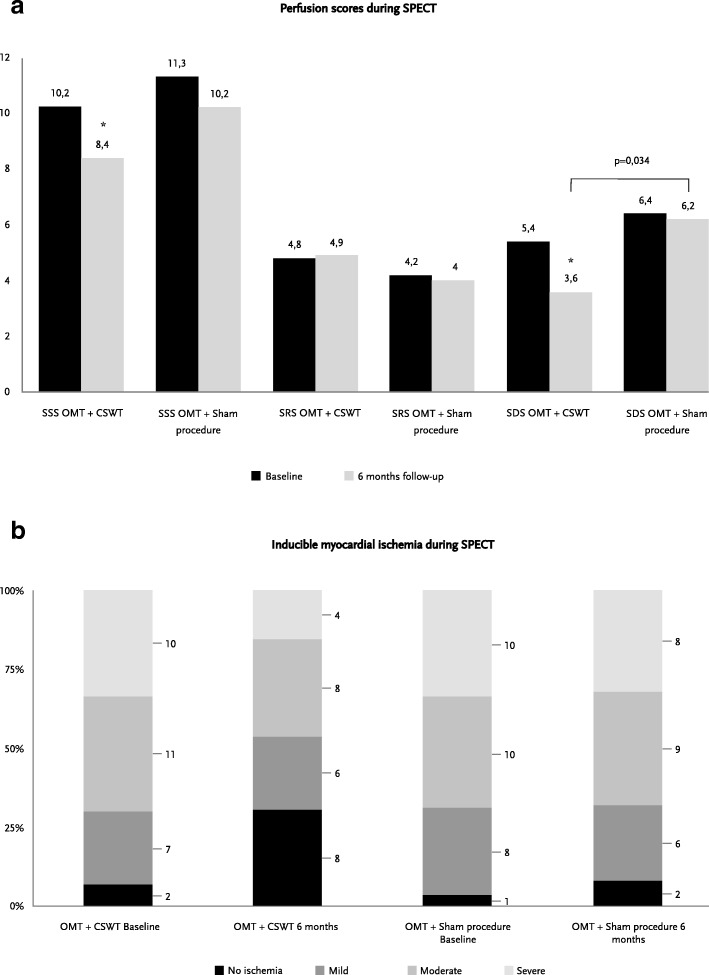
Dynamics of inducible myocardial ischemia evaluated by single photon emission computed tomography. **a** dynamics of perfusion scores at baseline, 3 and 6 months of follow up in CSWT and sham procedure group; **b** distribution of mild, moderate, severe or no ischemia at baseline, 3 and 6 months of follow up in CSWT and sham procedure group. CSWT – Cardiac shock wave therapy, OMT – optimal medical therapy, SSS – summed stress score, SRS – summed rest score, SDS – summed difference score. Moderate ischemia defined as summed difference score (SDS) at least 4. * - P was paired in the group and considered as significant (P < 0.05)

CSWT treatment caused a significant reduction of stress-induced ischemia in contrast to the sham applications at 3 months as demonstrated by the decrease of WMSI at stress (Table 2, Fig. 1) at the first time point of the follow-up. Positive anti-ischemic effect was maintained in the OMT + CSWT group throughout the study, however it also appeared in the OMT + sham procedure group at 6-months follow-up, resulting in no significant difference in the stress regional myocardial function between the groups at the end of the study (Table 2, Fig. 1). Analysis of the SPECT perfusion defects demonstrated beneficial effects of the CSWT treatment at 6-months follow-up. Significant reduction in the amount of reversible ischemia (decreased SDS) was achieved in the OMT + CSWT group and not the OMT + sham procedure group (Table 2, Fig. 2).

**Table 2:** The primary, secondary endpoints and other characteristics of the imaging tests

	OMT + CSWT group			OMT + sham procedure group		
	Baseline	3- month	6- month	Baseline	3- month	6- month
*Primary endpoints*						
WMSI at stress during DSE	1.6 ± 0.4	1.4 ± 0.4*	1.4 ± 0.3*	1.6 ± 0.4	1.5 ± 0.3	1.4 ± 0.3*
SDS during SPECT^#^	5.5 [3.3; 7.0]	–	3.0 [0; 5.0]*^, &^	4.0 [3.0; 9.0])	–	5.0 [3.0; 8.0]
*Secondary endpoints*						
WMS at stress during DSE	26.8 ± 7.0	24.2 ± 7.3*	23.1 ± 5.8*	26.3 ± 6.5	25.0 ± 5.7	23.5 ± 5.1*
LVEF at stress during DSE, %	49.8 ± 11.2	54.1 ± 12.3*	56.8 ± 9.4*	51.6 ± 11.0	53.4 ± 11.6	53.7 ± 9.7
Number of patients with at least moderate ischemia in DSE, n (%)	17 (57)	6 (21)*	5 (18)*	14 (48)	10 (36)	9 (35)
Number of patients with at least moderate ischemia in SPECT, n (%)	22 (73)	–	12 (46)*	21 (72.4)	–	17 (68)
ECG changes during stress DSE, n (%)	22 (73)	13 (46)*^, &^	13 (46)*	19 (66)	21 (75)	16 (62)
Angina during stress DSE, n (%)	23 (77)	12 (43)*	10 (36)*	18 (62)	13 (46)	11 (42)*
Global PSS at stress during DSE, %	−15.0 ± 3.2	−13.7 ± 3.0	−14.0 ± 2.3	−15.1 ± 4.5	− 13.7 ± 3.6	−13.6 ± 2.4
Summed stress score (SSS) during SPECT^#^	8.5 [5.3; 12.8]	–	5.0 [2.0; 12.0]*	10.0 [4.0; 15.0]	–	8.0 [3.0; 14.0]
TPD at stress during SPECT, %^#^	13.0 [6.3; 18.8]	–	7.0 [3.0; 17.5]*	15.0 [6.0; 22.0]	–	12.0 [4.0; 20.0]
TPD at rest during SPECT, %^#^	2.0 [0; 7.8]	–	0 [0; 7.0])	2.0 [0; 10.0])	–	3.0 [0; 9.0]
TPD difference during SPECT, %	7.0 [4.3; 11.0]	–	4.0 [0; 7.0]*^,&^	7.0 [5.0; 13.0]	–	7.0 [4.0; 12.0]
*Other tests characteristics*						
WMS at rest during DSE	23.4 ± 7.8	23.6 ± 7.8	22.6 ± 6.4	23.8 ± 7.0	24.3 ± 6.9	22.8 ± 5.7
WMSI at rest during DSE	1.4 ± 0.5	1.4 ± 0.5	1.3 ± 0.4	1.4 ± 0.4	1.4 ± 0.4	1.3 ± 0.3
LVEF at rest during DSE, %	46.5 ± 10.6	47.3 ± 11.0	49.8 ± 8.6*	48.5 ± 9.0	48.2 ± 8.6	48.5 ± 8.1
Number of positive DSE tests, n (%)	21 (70)	10 (36)*	8 (29)*	20 (69)	13 (46)	11 (4)
Number of positive SPECT tests, n (%)	28 (93)	–	18 (69)*	28 (96)	–	21 (84)
Global PSS at rest, %	−14.8 ± 3.4	−13.2 ± 3.8	−13.9 ± 2.7	−14.1 ± 2.2	−13.5 ± 2.6	−13.0 ± 1.9
SRS during SPECT^#^	1.0 [0; 5.8]	–	0 [0; 5.0]	2.0 [0; 7.0]		2.0 [0; 6.0]

*WMSI* wall motion score index, *SDS* summed difference score, *DSE* dobutamine stress echocardiography, *SPECT* single photon emission computed tomography, *SRS* summed rest score, *ECG* electrocardiogram, *CH* chamber view, *CSWT* Cardiac shock wave therapy, *LVEF* left ventricular ejection fraction, *OMT* optimal medical therapy, *PSS* peak systolic strain

Moderate ischemia was defined as ≥3 segments with stress induced severe hypokinesis or akinesis in DSE and as SDS 4–7 in SPECT

*- *P < 0.05,* comparison of follow up to baseline in the group, &- *P < 0.05,* comparison between study groups, # -values are presented as median [Interquartile range]

### Secondary endpoints of the study

Changes in WMS were equivalent to the dynamics of WMSI: the former decreased only in the interventional group in a short-term, but at the end of the study reached significant reduction in all study patients. Patients in the OMT + CSWT group demonstrated higher ejection fraction at stress at both follow-up time points as well as an increased rest LVEF at the end of study. In contrast, no increase was reported in the OMT + sham procedure group at any time point (Table 2, Fig. 1b). Number of patients with at least moderate ischemia significantly decreased at both assessment points only in OMT + CSWT group (Table 2, Fig. 1), as well as the occurrence of stress angina and ST depression (Table 2). The ECG changes were remarkably less frequent in the interventional group than in sham procedure group at 3 months.

Reduced baseline global peak systolic strain (PSS) was found in all patients both at rest and during stress (Table 2). CSWT treatment demonstrated a protective effect on myocardial deformation throughout the study period: strain values did not significantly change in the OMT + CSWT group in contrast to the OMT + sham procedure group. At 6-months follow up a significant decrease of rest 2-chamber view PSS (− 15.1 ± 3.3 to − 13.3 ± 2.1, *p* = 0.026) and of stress 4-chamber view PSS (− 15.3 ± 4.8 to − 12.9 ± 2.5, *p* = 0.002) was recorded in the latter group. Finally, a significant reduction of myocardial hypoperfusion assessed by decreased SSS, as well as a reduction in the stress TPD was achieved in the OMT + CSWT group and not the OMT + sham procedure group (Table 2, Fig. 2) at the end of the study.

At 6-months follow-up number of patients with no induced ischemia increased significantly in OMT + CSWT group compared OMT + sham procedure group [8 (30.8%) vs. 2 (8%), *p* = 0.042]. Patients with moderate to severe inducible myocardial ischemia decreased to 12 (46.2%) in OMT + CSWT group and 17 (68%) in OMT + sham procedure group (*p* = 0.296) (Table 2, Fig. 2).

### Summary effect of CSWT treatment

**Fig. 3:**
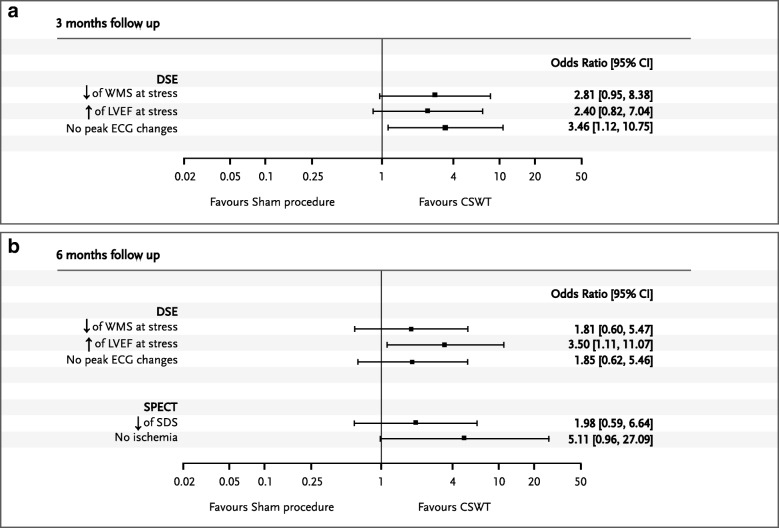
Summarized treatment effects of the cardiac shock wave therapy compared with sham procedure. **a** at 3 months of follow up; **b** – at 6 months of follow up. DSE – dobutamine stress echocardiography, ECG – electrocardiogram, LVEF – left ventricle ejection fraction, SDS – summed difference score, SPECT - single photon emission computed tomography, WMS – wall motion score

The summary effect of CSWT compared to sham procedure on endpoint imaging parameters is shown in Fig. 3. The addition of CSWT to OMT resulted in effective reduction of established ischemia signs compared to OMT alone and more frequent normalization of myocardial perfusion and contraction during stress.

### Reproducibility of DSE and SPECT parameters

The perfusion scores and rest ultrasound LVEF showed excellent reproducibility, followed by good reproducibility of WMS values (Table 3). Estimation of longitudinal deformation marker was the most variable among study endpoints.

**Table 3:** Reproducibility of the primary and secondary DSE and SPECT parameters

	Mean difference ± SD	Inter-observer ICC	95% CI	*P* value
WMS at stress during DSE	−1.7 ± 4.6	0.816	(0.54, 0.93)	< 0.001
SDS during SPECT	0.73 ± 3.4	0.757	(0.64, 0.84)	< 0.001
LVEF at stress during DSE, %	3.8 ± 8.3	0.774	(0.45, 0.92)	< 0.001
Global PSS stress, %	−2.0 ± 2.6	0.602	(0.13, 0.85)	0.009
SSS during SPECT	0.01 ± 3.1	0.950	(0.92, 0.97)	< 0.001
WMS at rest during DSE	−0.2 ± 4.1	0.861	(0.64, 0.95)	< 0.001
LVEF at rest during DSE, %	1.3 ± 4.6	0.932	(0.81, 0.98)	< 0.001
SRS during SPECT	−0.57 ± 2.9	0.942	(0.91, 0.96)	< 0.001
Global PSS rest, %	−1.02 ± 1.8	0.625	(0.14, 0.87)	0.008

*CI* confidence interval, *ICC* interclass correlation coefficient, *LV* left ventricular, *PSS* peak systolic strain, *SD* standard deviation

## Discussion

As a part of the recently published randomized, triple-blind, sham procedure-controlled trial [16], we performed an imaging sub-study that evaluated the capacity of the CSWT to reduce myocardial ischemia determined by DSE and SPECT tests. Current publications provide limited information about the effects of CSWT on global and regional myocardial function and perfusion, therefore our study brings novel high-quality data on the effects of this promising method on objective signs of myocardial ischemia. Both primary sub-study endpoints have changed significantly at the end of the study, though only perfusion score was different in the intervention group.

The analysis of the DSE data revealed that CSWT improved regional myocardial contractility and LV ejection fraction during stress. Due to particular study design (i.e. repetitive DSE testing at 3 and 6 months after the treatment initiation), we were able to demonstrate a remarkable reduction in stress induced ischemia assessed by semi-quantitative WMSI, **or WMS,** and an improvement of LVEF at 3 months, which was significant only in CSWT + OMT group. This early improvement of myocardial function during stress confirms the beneficial effect of shock acoustic waves, which may be attributed to angiogenetic and vasoactive mechanisms. The positive effect on regional myocardial function was maintained further until the end of study at 6 months after the CSWT initiation, along with markedly higher LVEF not only during stress, but also at rest. This at least partially may be explained by enhanced coronary circulation due to the intervention. To our knowledge, this is the first study that evaluated the effects of CSWT on LVEF during DSE test.

Importantly, the improvement in imaging endpoints of WMSI/WMS, LVEF and perfusion scores were corroborated by decrease in the number of patients with at least moderate ischemia, ECG changes and angina during stress, which was significant only in CSWT group, except angina at 6 months.

Though blind randomization was performed using random number table, by the play of chance a history of myocardial infarction was documented more often in the sham procedure group (23 vs 15); however, it did not produce the difference in rest WMS or LVEF (Table 2). Positive changes of the alleviation of myocardial ischemia after 6 months were also achieved in the control group. We interpret it as the result of the optimization of the medical treatment, which in the course of the trial can frequently be more effective compared to routine practice. The design of the study resulted in repeated appointments between the study patients and a cardiologist, potentially increasing the compliance to prescriptions.

For the assessment of myocardial mechanics at rest and during stress, we utilized not only visual assessment but also innovative markers of deformation imaging. Previous CSWT studies analysed changes of peak systolic strain rate [13, 31]. The results showed significant increase of PSSR at 6 and 12 months follow-up in CSWT group compared with controls, accompanied by significant increase in the amplitude of regional myocardial motion in M-mode [13, 31]. We did not find any previous reports of systolic strain dynamics in CSWT trials. The purpose of inclusion of these objective functional parameters was to register probable subtle differences in contractility in the course of the treatment, which sometimes cannot be seen by the naked eye. We found that the application of SWs to all LV segments had a protective effect on myocardial deformation: peak systolic strain values remained unchanged in the intervention group in contrast to the sham procedure group, where global PSS decreased significantly at the end of the study. This important finding suggests that CSWT might inhibit the progression of systolic dysfunction and ventricular remodelling.

Myocardial perfusion imaging results demonstrated that the adjunct of CSWT to the OMT results in a significant reduction of ischemia as compared to the OMT alone. The complete normalization in perfusion scores was more common in patients assigned to OMT + CSWT group. As a method associated with radiation exposure and being more resource-consuming, SPECT was not repeated at 3 months time-point. Our results are in agreement with previous studies that demonstrated the ability of CSWT to improve myocardial perfusion in patients with refractory angina. Higher microvascular density and upregulation of vascular endothelial grow factor, Fms-related tyrosine kinase 1 and placental grow factor were documented as a result of shock wave application in a rodent model [32]. Alunni et al. demonstrated a significant reduction of mean SSS from 21.3 ± 10.3 to 14.1 ± 10.1 (*p* = 0.003) compared with baseline, but SPECT was not performed in controls at follow-up [6]. Kazmi et al. reported larger numbers of patients with reduced severity of ischemia at follow-up [11]. However, our study is the first to evaluate the effects of CSWT on local perfusion using SPECT and comp.

aring treatment groups in a triple blind and randomized manner.

The anti-ischemic effect of CSWT was clearly proven by cardiac imaging techniques, as well as by symptoms and ECG changes during stress in the present triple blind, randomized placebo-controlled trial. In our main study [16], which assessed the efficacy of CSWT on exercise duration and angina symptoms in addition to OMT in patients with objective evidence of myocardial ischemia, we revealed a neutral result of CSWT on the exercise duration during treadmill stress test, as well as on angina symptoms, angina class, nitroglycerine consumption and quality of life. Interestingly, recent ORBITA trial failed to show symptomatic benefit of percutaneous coronary intervention for stable angina patients compared to sham treatment [33]. Both groups had significant clinical improvement in angina symptoms and exercise variables. Total exercise duration was selected as primary endpoint similar to our main study and did not differ between groups 6 weeks after intervention. Of note, only peak dobutamine WMSI improved significantly with the intervention, similarly to our study.

Our study had a few limitations. First, no detailed analysis on the CSWT responders and non-responders was performed, and it remains a target for the future studies. Second, some differences in the ultrasound image quality of the enrolled patients, e.g. after CABG, having overweight, could have affected the accuracy of the WMSI analysis. Despite these limitations, it is the only study to date to evaluate the effects of CSWT on myocardial function and perfusion using stress imaging techniques and comparing treatment groups in a blind randomized, placebo-controlled manner.

## Conclusions

The results of the prospective randomized imaging substudy suggest that cardiac shock wave therapy effectively improves myocardial function and perfusion in stable angina patients. Target patient population which could mostly benefit from such kind of intervention has yet to be defined.

## Data Availability

The datasets generated and analysed during the current study are available from principal investigator Jelena Čelutkienė and Greta Burneikaitė on reasonable request.

## Abbreviations

CAD: Coronary artery disease
CCS: Canadian Cardiac Society
CSWT: Cardiac shock wave therapy
DSE: Dobutamine stress echocardiography
ECG: Electrocardiogram
EF: Ejection fraction
LV: Left ventricle
MPI: Myocardial perfusion imaging
NYHA: New York Heart Association
OMT: Optimal medical treatment
PSS: Peak systolic strain
SDS: Summed difference score
SPECT: Single photon emission computed tomography
SRS: Summed rest score
SSS: Summed stress score
SW: Shock waves
TPD: Total perfusion defect
WMS: Wall motion score
WMSI: Wall motion score index

## References

[1] Andréll P, Ekre O, Grip L, Währborg P, Albertsson P, Eliasson T (2011). Fatality, morbidity and quality of life in patients with refractory angina pectoris. Int J Cardiol Elsevier.

[2] Williams B, Menon M, Satran D, Hayward D, Hodges JS, Burke N (2010). Patients with coronary artery disease not amenable to traditional revascularization: prevalence and 3-year mortality. Catheter Cardiovasc Interv.

[3] Gotte G, Amelio E, Russo S, Marlinghaus E, Musci G, Suzuki H (2002). Short-time non-enzymatic nitric oxide synthesis from L-arginine and hydrogen peroxide induced by shock waves treatment. FEBS Lett.

[4] Ciampa AR, de Prati AC, Amelio E, Cavalieri E, Persichini T, Colasanti M (2005). Nitric oxide mediates anti-inflammatory action of extracorporeal shock waves. FEBS Lett.

[5] Fu M, Sun C-K, Lin Y-C, Wang C-J, Wu C-J, Ko S-F (2011). Extracorporeal shock wave therapy reverses ischemia-related left ventricular dysfunction and remodeling: molecular-cellular and functional assessment. PLoS One.

[6] Alunni G, Marra S, Meynet I, D’amico M, Elisa P, Fanelli A (2015). The beneficial effect of extracorporeal shockwave myocardial revascularization in patients with refractory angina. Cardiovasc Revasc Med.

[7] Zuoziene G, Leibowitz D, Celutkiene J, Burneikaite G, Ivaskeviciene L, Kalinauskas G (2015). Multimodality imaging of myocardial revascularization using cardiac shock wave therapy. Int J Cardiol.

[8] Schmid J-P, Capoferri M, Wahl A, Eshtehardi P, Hess OM (2013). Cardiac shock wave therapy for chronic refractory angina pectoris. A prospective placebo-controlled randomized trial. Cardiovasc Ther.

[9] Prasad M, Wan Ahmad WA, Sukmawan R, Magsombol E-BL, Cassar A, Vinshtok Y (2015). Extracorporeal shockwave myocardial therapy is efficacious in improving symptoms in patients with refractory angina pectoris – a multicenter study. Coron Artery Dis.

[10] Kikuchi Y, Ito K, Ito Y, Shiroto T, Tsuburaya R, Aizawa K (2010). Double-blind and placebo-controlled study of the effectiveness and safety of extracorporeal cardiac shock wave therapy for severe angina pectoris. Circ J.

[11] Kazmi WH, Rasheed SZ, Ahmed S, Saadat M, Altaf S, Samad A (2012). Noninvasive therapy for the management of patients with advanced coronary artery disease. Coron Artery Dis.

[12] Leibowitz D, Weiss AT, Rott D, Durst R, Lotan C (2013). The efficacy of cardiac shock wave therapy in the treatment of refractory angina: a pilot prospective, randomized, double-blind trial. Int J Cardiol.

[13] Nirala S, Wang Y, Peng Y-Z, Yang P, Guo T (2016). Cardiac shock wave therapy shows better outcomes in the coronary artery disease patients in a long term. Eur Rev Med Pharmacol Sci.

[14] Yang P, Guo T, Wang W, Peng Y-Z, Wang Y, Zhou P (2013). Randomized and double-blind controlled clinical trial of extracorporeal cardiac shock wave therapy for coronary heart disease. Heart Vessel.

[15] Burneikaitė G, Shkolnik E, Čelutkienė J, Zuozienė G, Butkuvienė I, Petrauskienė B (2017). Cardiac shock-wave therapy in the treatment of coronary artery disease: systematic review and meta-analysis. Cardiovasc Ultrasound.

[16] Shkolnik E, Burneikaitė G, Jakutis G, Scherbak M, Zuozienė G, Petrauskienė B (2018). A randomized, triple-blind trial of cardiac shock-wave therapy on exercise tolerance and symptoms in patients with stable angina pectoris. Coron Artery Dis Coronary Artery Disease.

[17] Moher D, Hopewell S, Schulz KF, Montori V, Gøtzsche PC, Devereaux PJ (2010). CONSORT 2010 explanation and elaboration: updated guidelines for reporting parallel group randomised trials. BMJ..

[18] Shkolnik E, Burneikaite G, Celutkiene J, Scherbak M, Zuoziene G, Petrauskiene B, et al. Efficacy of cardiac shock wave therapy in patients with stable angina : The design of randomized , triple blind , sham-procedure controlled study. Anatol J Cardiol. 2018;19(2):100–109. 10.14744/AnatolJCardiol.2017.8023 PMC5864803 29424731

[19] Montalescot G, Sechtem U, Achenbach S, Andreotti F, Arden C, Task Force Members (2013). 2013 ESC guidelines on the management of stable coronary artery disease. Eur Heart J.

[20] Sicari R, Nihoyannopoulos P, Evangelista A, Kasprzak J, Lancellotti P, Poldermans D (2008). Stress echocardiography expert consensus statement: European Association of Echocardiography (EAE) (a registered branch of the ESC). Eur J Echocardiogr.

[21] Hesse B, Tägil K, Cuocolo A, Anagnostopoulos C, Bardiés M, Bax J (2005). EANM/ESC procedural guidelines for myocardial perfusion imaging in nuclear cardiology. Eur J Nucl Med Mol Imaging.

[22] Cerqueira MD, Weissman NJ, Dilsizian V, Jacobs AK, Kaul S, Laskey WK (2002). Standardized myocardial segmentation and nomenclature for tomographic imaging of the heart. A statement for healthcare professionals from the cardiac imaging Committee of the Council on Clinical Cardiology of the American Heart Association. Circulation.

[23] Shaw LJ, Berman DS, Picard MH, Friedrich MG, Kwong RY, Stone GW (2014). Comparative definitions for moderate-severe ischemia in stress nuclear, echocardiography, and magnetic resonance imaging. JACC Cardiovasc Imaging.

[24] Cerqueira MD, Verani MS, Schwaiger M, Heo J, Iskandrian AS (1994). Safety profile of adenosine stress perfusion imaging: results from the Adenoscan multicenter trial registry. J Am Coll Cardiol.

[25] Berman D, Abidov A, Kang X, Hayes SW, Friedman JD, Sciammarella MG (2004). Prognostic validation of a 17-segment score derived from a 20-segment score for myocardial perfusion SPECT interpretation*1. J Nucl Cardiol.

[26] Hachamovitch R, Berman DS, Kiat H, Cohen I, Cabico JA, Friedman J (1996). Exercise myocardial perfusion SPECT in patients without known coronary artery disease: incremental prognostic value and use in risk stratification. Circulation..

[27] Hachamovitch R, Hayes SW, Friedman JD, Cohen I, Berman DS (2003). Comparison of the short-term survival benefit associated with revascularization compared with medical therapy in patients with no prior coronary artery disease undergoing stress myocardial perfusion single photon emission computed tomography. Circulation..

[28] Popović ZB, Thomas JD (2017). Cardiovascular diagnosis and therapy. Vol. 7, cardiovascular diagnosis and therapy.

[29] Koo TK, Li MY (2016). A guideline of selecting and reporting Intraclass correlation coefficients for reliability research. J Chiropr Med Elsevier.

[30] Portney LG, Watkins MP (2000). Foundations of clinical research: applications to practice, 2nd Edition.

[31] Wang Y, Guo T, Ma T, Cai H, Tao S, Peng Y (2012). A modified regimen of extracorporeal cardiac shock wave therapy for treatment of coronary artery disease. Cardiovasc Ultrasound BioMed Central.

[32] Zimpfer D, Aharinejad S, Holfeld J, Thomas A, Dumfarth J, Rosenhek R (2009). Direct epicardial shock wave therapy improves ventricular function and induces angiogenesis in ischemic heart failure. J Thorac Cardiovasc Surg Elsevier.

[33] Al-Lamee R, Thompson D, Dehbi H-M, Sen S, Tang K, Davies J (2018). Percutaneous coronary intervention in stable angina (ORBITA): a double-blind, randomised controlled trial. Lancet.

